# Psychological safety is associated with better work environment and lower levels of clinician burnout

**DOI:** 10.1093/haschl/qxae091

**Published:** 2024-07-17

**Authors:** Rosalind de Lisser, Mary S Dietrich, Joanne Spetz, Rangaraj Ramanujam, Jana Lauderdale, Deonni P Stolldorf

**Affiliations:** School of Nursing, Vanderbilt University, Nashville, TN 37240, United States; School of Nursing, University of California San Francisco, San Francisco, CA 94143, United States; School of Nursing, Vanderbilt University, Nashville, TN 37240, United States; Department of Biostatistics, Vanderbilt University Medical Center, Nashville, TN 37232, United States; Philip R. Lee Institute for Policy, University of California San Francisco, San Francisco, CA 94158, United States; Owen Graduate School of Management, Vanderbilt University, Nashville, TN 37203, United States; School of Nursing, Vanderbilt University, Nashville, TN 37240, United States; School of Nursing, Vanderbilt University, Nashville, TN 37240, United States

**Keywords:** occupational burnout, psychological safety, health care environment, quality measurement

## Abstract

Burnout is attributed to negative work environments and threatens patient and clinician safety. Psychological safety is the perception that the work environment is safe for interpersonal risk-taking and may offer insight into the relationship between the work environment and burnout. In this cross-sectional analysis of survey data from 621 nurse practitioners in California, we found that one-third (34%) experienced high burnout. Four factors in the work environment were negatively associated with burnout and positively associated with psychological safety. Significant mediation effects of psychological safety were observed on the relationships between each work environment factor and both emotional exhaustion and depersonalization. The largest mediation effects were observed on the total effects of Nurse Practitioner–Physician Relations and Practice Visibility on Emotional Exhaustion (37% and 32%, respectively) and Independent Practice and Support and NP-Administration Relations on Depersonalization (32% and 29%, respectively). We found, overall, that psychological safety decreased the strength of the negative relationship between work environment and burnout. We argue that research, practice, and policy efforts to mitigate burnout and improve the work environment should consider psychological safety as a metric for system-level well-being.

## Introduction

Burnout is associated with turnover,^1^ increased mortality,^2^ and rising costs.^3^ Burnout is widespread and driven by factors in the work environment, such as lack of support^4^ and limited control over work responsibilities.^5^ Health care work environments are complex, hierarchical, and chaotic, demanding communication and task integration among clinicians and non-clinicians to achieve safe and successful outcomes.^5^ The health care work environment is complex and defined by overlapping factors involving tasks, social context, and organizational culture, depending on role and position.^6^ For nurses, the work environment is defined as “organizational characteristics of the work setting which enable or constrain nursing practice,”^7^ a definition expanded for nurse practitioners (NPs) to include the manner in which the “organization interacts with NPs, affecting their behavior and outcomes.”^8^ The NP work environment includes structural factors such as policies, procedures, and organizational norms, which promote or inhibit success; this is in addition to relational factors such as interpersonal relationships, communication, and teamwork.^9^ While these factors are not unique to NPs, they may be experienced differently depending on social status in the organization and contribute to a culture of hierarchy defined by power differentials between professional groups.^10^ Nurse practitioners are uniquely situated in health care, as advanced-practice nurses their work environment is influenced by structural policies at the federal, state, and organizational level that influence scope of practice (SOP), autonomy, and agency at work.^9^ The NP workforce is expanding rapidly and occupies an important role in the delivery of health care; yet, NP autonomy, roles and responsibilities, and outcomes are hindered by variabilities in physician supervision requirements,^11^ insufficient work-related support,^12^ and devaluation of the NP role by administrators and physicians.^13^ Such challenges in the work environment contribute to strained interpersonal relationships between NPs and physicians and administrators,^14^ which may be influenced by the presence of psychological safety.

Psychological safety is an interpersonal construct, critical to organizational learning and teamwork. It is defined as the perception that one's work environment is “safe from threat and tolerates failure without retaliation.”^15^ Psychologically safe workplaces support and value “speaking up” behaviors necessary for learning and innovation at work.^16,17^ Studies show that psychological safety is associated with error reporting,^18^ caring work environments,^19^ and decreased burnout.^20^

Given the complexities of the NP work environment, this study expands on past work linking the NP work environment with burnout,^21^ by exploring the mediating role of psychological safety in the relationship between factors in the NP work environment and burnout.

## Data and methods

This study was reviewed and approved by the institutional review boards (IRB) of the University of California San Francisco and Vanderbilt University (IRB #22-36261 and IRB #221600, respectively). We followed Strengthening the Reporting of Observational Studies in Epidemiology (STROBE) reporting guidelines for cross-sectional studies.^22^

### Study design and setting

This cross-sectional study was a secondary analysis of a larger NP workforce survey conducted in California using validated questionnaires via electronic and paper-based surveys (see Appendix 1 for a full description). The 2022 Survey of California Nurse Practitioners and Nurse Midwives sought to examine the practice environment of NPs in California, before enactment of legislation (January 2023) that created a pathway to NP independent practice.^23^

### Participants and sample size

Participants were selected from those who completed the 2022 Survey of California Nurse Practitioners and Nurse Midwives (*n* = 993). Inclusion criteria for this study were current employment in a position requiring NP licensure and completion of at least 70% of items^24^ on each outcome measure of interest. Data were not weighted and did not seek to be representative of the population.

### Data collection

Data were collected from July 2022 to February 2023. Prospective respondents were recruited by email or US postal service and provided with information about the study, voluntary participation, confidentiality, and data security measures. Completion of the survey indicated informed consent. In addition to the outcome measures described below, covariates including demographic characteristics, work setting, job tenure, and hours worked in the past week were assessed.

### Survey measures

#### Burnout

Burnout was measured using the 9-item Emotional Exhaustion (EE) and 5-item Depersonalization (DE) subscales of the Maslach Burnout Inventory Human Services Survey (MBI-HSS).^25^ Items are assessed on a 7-point scale from 0 (never) to 6 (every day), with higher scores indicating more severe burnout (see Appendix 2 for sample questions). The scales' content and construct validity have been previously established for use in nurses and physicians.^26,27^ Cronbach alpha coefficients in this study were .94 (EE) and .80 (DE). A categorical indicator of burnout was also generated for the EE subscale, using a score of ≥27, which indicates high levels of burnout.^28,29^

#### Psychological safety

Edmonson's 7-item Psychological Safety Scale^30^ was used to assess perceived psychological safety. Participants were asked to rate their degree of agreement about the team that they work with most on a 6-point scale from 1 (strongly disagree) to 6 (strongly agree), with higher scores indicative of higher perception of psychological safety (see Appendix 2 for sample items and scoring). The internal consistency from prior research ranged from 0.67 to 0.77 and included samples of nurses.^19,31^ Consistent with those values, the Cronbach alpha coefficient score in this study was .75.

#### Work environment

The Nurse Practitioner Primary Care Organizational Climate Questionnaire measures 4 domains of the work environment.^32^ The scale has been validated for use in the primary care^33^ and acute care environments^34^ and sample subscale items can be found in Appendix 2. Each item was rated on a 4-point Likert scale from 1 (strongly disagree) to 4 (strongly agree); thus, higher scores are indicative of better conditions. Consistent with previously published reliability (.87–.94),^33^ our Cronbach alpha coefficients for each scale were Practice Visibility = .87, Independent Practice and Support [IPS] = .98, NP–Administration Relations [NP-AR] = .91, and NP–Physician Relations [NP-PR] = .82.

### Statistical methods

IBM SPSS version 29.0 statistics software^35^ was used for data analysis. We used frequency distributions to summarize the categorical variables and, due to skewness of most continuous variable distributions, medians and IQRs were reported. Bootstrapped 95% CIs were generated around the observed median values for each key study variable. While completion of those key study measures was required for inclusion in the sample, a small percentage (0.48%–1.93%) of the covariates (setting, job tenure, hours worked) was missing. Those missing values were imputed using the Markov chain Monte Carlo algorithm in SPSS.

All key study variables and continuous covariate data distributions were transformed to normalize them prior to inclusion in subsequent analyses with parametric assumptions. Practice setting differences in reports of psychological safety, burnout (EE and DE), and work environment were assessed using analysis of variance. We conducted post hoc tests of statistically significant findings using Dunnett's C criteria and Pearson coefficients to assess the strength and direction of correlations among job tenure and hours worked per week with each of the study measures, as well as intercorrelations among outcome measures. The PROCESS version 4.3 macro^36^ in SPSS was used to examine the mediation effect of psychological safety on the direct effects between the 4 work environment factors and the 2 EE and DE indicators of burnout. Each model included practice setting, job tenure, and hours worked per week as covariates. Multicollinearity among outcome measures and covariates was assessed prior to conducting the mediation analyses. Regardless of the model, all tolerance coefficients were >0.7. Bootstrapped 95% CIs were generated around each of the parameter estimates from the PROCESS models. Interpretations of statistical significance used *P* < .05.

## Results

### Participant characteristics

A total of 757 participants responded to the California statewide survey and were currently working in a position requiring NP licensure; of those, 621 (82%) completed the measures necessary for this analysis. With the exception of practice setting, no significant differences in demographic characteristics were observed between those included and the 136 excluded due to missing data (*P* > .20). Compared with the analysis sample, a higher percentage of those excluded were working in a location other than ambulatory care, hospitals, or long-term care (30% vs 18%, *P* = .002). The analysis sample was predominantly female (89.7%), the mean age was 50 (SD ±12) years, and a majority identified as Caucasian/White/European/Middle Eastern (64%). Average years licensed as an NP was 13.7 (SD ±10) and a majority (61.5%) reported being on the job for <5 years (median = 4.3, IQR = 1.7–9.1). Most worked a median of 40 hours per week and 13% worked >40 hours. Aligned with national workforce data,^37^ approximately half worked in ambulatory care (48%), followed by hospitals or medical centers (30%) and long-term care (3.8%) (Table 1).

**Table 1.** Demographic characteristics of the California nurse practitioner sample.

	Study sample, *n* (%), [95% CI]
Gender (*n* = 614)	
Female	551 (89.7), [87.0, 92.0]
Male	60 (9.8), [7.6, 12.4]
Transgender, nonbinary, genderqueer	3 (0.5), [0.1, 1.5]
Age (*n* = 621)	
25–24 y	52 (8.4), [6.4, 11.0]
35–44 y	181 (29.1), [25.6, 33.0]
45–54 y	160 (25.8), [22.4, 30.0]
55–64 y	137 (22.1), [18.9, 26.0]
65+ y	91 (14.7), [12.0, 18.0]
Race and ethnicity (*n* = 616)	
African American/Black/African	30 (4.9), [3.4, 7.1]
American Native Indian/Alaskan Native	8 (1.3), [0.6, 2.6]
Asian/Pacific Islander	128 (20.8), [17.5, 25.0]
Caucasian/White/European/Middle Eastern	397 (64.4), [60.5, 69.0]
Latino Hispanic	35 (5.7), [4.0, 8.0]
Mixed/other	18 (2.9), [1.7, 5.0]
Licensed as a nurse practitioner (*n* = 597)	
1–5 y	148 (24.8), [21.5, 29.0]
6–10 y	144 (24.1), [20.7, 28.8]
11–20 y	147 (24.6), [21.2, 29.0]
21+ y	158 (26.5), [23.0, 31.0]
Job tenure (*n* = 615)	
<1 y	89 (14.5), [11.0, 18.0]
1–2 y	152 (24.7), [21.4, 29.0]
3–5 y	137 (22.3), [19.1, 26.0]
6–10 y	117 (19.0), [16.1, 23.0]
11+ y	120 (19.5), [16.5, 23.0]
Work per week (*n* = 618)	
<40 h	285 (46.1), [42.2, 51.0]
40 h	252 (40.8), [36.8, 45.0]
>40 h	81 (13.1), [10.5, 17.0]
Practice setting (*n* = 609)	
Hospital or medical center	182 (29.9), [26.2, 34.0]
Ambulatory care setting	295 (48.4), [44.3, 53.0]
Long-term care and home health	23 (3.8), [2.4, 6.0]
Other^a^	109 (17.9), [15.0, 22.0]

Abbreviations: No, number; CI, confidence interval.

^a^Correctional system, academic education program.

### Burnout, the work environment, and psychological safety

Approximately one-third (*n* = 210, 34%) of participants experienced high burnout, with scores ≥27 on the EE scale of the MBI-HSS.^27^ The median MBI EE and DE scores were 2.2 and 0.6, respectively (range, 0–6). Hours worked per week was positively correlated with both EE and DE scores(*r* = 0.17 and 0.13 respectively, *P* ≤ .002). Compared with NPs working in acute care, NPs in ambulatory care settings had significantly higher EE scores but did not differ on DE (median = 3.0 vs 3.3, respectively; Bonferroni*-*corrected, *P* < .05) (Appendix 3).

The 4 work environment subscales ranged from lowest for NP-AR (median = 2.9) to highest for IPS (median = 3.3), as described in Appendix 3. Job tenure was positively correlated with IPS and NP-PR (*r* = 0.15 and 0.20, respectively; *P* < .001). Nurse practitioners working in ambulatory care settings reported significantly higher IPS scores than NPs working in hospitals or medical centers (median = 3.4 vs 3.1, respectively; Bonferroni*-*corrected, *P* < .05). The median Psychological Safety Scale score was 4.6 (IQR, 3.8–5.2) and associated with job tenure (*r* = 0.17, *P* < .001), however not with job setting or work hours.

All correlations among outcome measures were significant (*P* < .001). The 4 factors in the work environment were inversely correlated with EE and DE and are presented in Table 2. The strongest negative associations with EE and DE were between NP-AR (*r* = −0.37 and −0.24) and IPS (*r* = −0.30 and *r* = −0.23). Psychological safety was inversely correlated with EE and DE burnout measures (*r* = −0.31 and *r* = −0.26) and positively correlated with all work environment factors. The strongest correlations with psychological safety were NP-PR (*r* = 0.44) and IPS (*r* = −0.40).

**Table 2.** Correlations among primary study measures.

	Work environment^a^			Psychological safety^f^	Burnout^g^	
	IND practice and support^b^	NP-ADM relations^c^	NP–physician relations^d^	Psychological safety	Emotional exhaustion	Depersonalization
Practice Visibility**^e^**	0.71	0.73	0.58	0.35	−0.25	−0.16
Independent Practice and Support**^b^**		0.67	0.67	0.40	−0.30	−0.23
NP–ADM Relations**^c^**			0.65	0.39	−0.37	−0.24
NP–Physician Relations**^d^**				0.44	−0.28	−0.19
Psychological Safety					−0.31	−0.26
Emotional Exhaustion						0.66

*n* = 621. Note all Pearson correlation coefficients have *P* values <.001.

Abbreviations: ADM, administration; IND, independent; NP, nurse practitioner.

^a^Nurse Practitioner Primary Care Organizational Climate Questionnaire.

^b^IND Practice and Support: perception of practice autonomy and independence in clinical decision-making.

^c^NP–Administration Relations: degree to which the organization and administration value the NP clinician.

^d^NP–Physician Relations: perception of degree to which physicians trust and value the NP.

^e^Practice Visibility: degree of NP role clarity and role understanding within the organization and administration.

^f^Psychological Safety Scale.

^g^Maslach Burnout Inventory Human Services Survey.

### Mediating effects of psychological safety on emotional exhaustion (EE)

After controlling for practice setting, job tenure, and hours worked, the unmediated total effects of the work environment scores with EE scores were all inverse and ranged from −0.25 (Practice Visibility [PV]) to −0.38 (NP-AR), as shown in Table 3. The statistically significant mediating effect of psychological safety was strongest for the association between NP-PR and EE (beta = −0.10, *P* < .05) (depicted in Appendix 4) The magnitude of that mediation effect represents a 37% reduction in the unmediated effect of −0.27. Psychological safety also reduced the total effect of PV on EE by 32% and reduced IPS by 27% (Table 3). The strongest unmediated total effect with EE was observed for NP-AR (*r* = −0.38), resulting in the smallest proportion of the effect (18%) mediated by psychological safety.

**Table 3.** Mediation effect of psychological safety on the direct effects of work environment with emotional exhaustion (n = 621).

	Coefficient	*P*-value	95% CI lower	95% CI upper	% Total effect^a^
Practice Visibility—X; Psychological Safety—M; Emotional Exhaustion—Y					
Total Effect (X → Y)	−0.25	0.001	−0.33	−0.17	
Mediated Direct Effect (X → Y)	−0.17	0.001	−0.26	−0.08	
**Indirect Effect (X →M→ Y)**^a^	**−0.08**	**0.001**	**−0.12**	**−0.04**	**32%** ^b^
X → M (Path a)	0.34	0.001	0.25	0.42	
M → Y (Path b)	−0.24	0.001	−0.33	−0.15	
Independent Practice and Support—X; Psychological Safety—M; Emotional Exhaustion—Y					
Total Effect (X → Y)	−0.30	0.001	−0.38	−0.22	
Mediated Direct Effect (X → Y)	−0.22	0.001	−0.30	−0.13	
**Indirect Effect (X →M→ Y)**^a^	**−0.08**	**0.001**	**−0.13**	**−0.04**	**26%** ^b^
X → M (Path a)	0.38	0.001	0.30	0.46	
M → Y (Path b)	−0.22	0.001	−0.30	−0.13	
NP Administration Relations—X; Psychological Safety—M; Emotional Exhaustion—Y					
Total Effect (X → Y)	−0.38	0.001	−0.46	−0.31	
Mediated Direct Effect (X → Y)	−0.32	0.001	−0.40	−0.23	
**Indirect Effect (X →M→ Y)**^a^	**−0.07**	**0.001**	**−0.11**	**−0.03**	**18%** ^b^
X → M (Path a)	0.38	0.001	0.29	0.47	
M → Y (Path b)	−0.18	0.001	−0.26	−0.10	
NP Physician Relations—X; Psychological Safety—M; Emotional Exhaustion—Y					
Total Effect (X → Y)	−0.27	0.001	−0.35	−0.19	
Mediated Direct Effect (X → Y)	−0.18	0.001	−0.26	−0.08	
**Indirect Effect (X →M→ Y)**^a^	**−0.10**	**0.001**	**−0.14**	**−0.05**	**37%** ^b^
X → M (Path a)	0.42	0.001	0.34	0.50	
M → Y (Path b)	−0.23	0.001	−0.32	−0.14	

Abbreviation: NP, nurse practitioner.

All effects adjusted for practice setting, hours worked, and job tenure.

^a^The indirect effect is the key effect of interest. It indicates how much of the total effect of the respective work environment factor on emotional exhaustion is due to the mediating effect of psychological safety.

^b^Proportion of the total effect that is mediated by psychological safety (indirect effect/total effect).

### Mediating effects of psychological safety on depersonalization (DE)

The total unmediated effect of factors in the work environment on DE was smaller than effects observed for EE (beta ranging from −0.15 for PV to −0.24 for NP-AR), described in Table 4. The mediating effects of psychological safety were comparable to effects observed for EE (−0.07 to −0.09, *P* < .05). Yet, because the total effects were smaller, the proportional reduction by psychological safety was greater, ranging from 29% for NP-AR to 32% for IPS (depicted in Appendix 4). After accounting for psychological safety, the mediated direct effects of PV and NP-PR on DE were no longer statistically significant (*P* ≥ .05) (Table 4).

**Table 4.** Mediation effect of psychological safety on the direct effects of work environment with depersonalization (n = 621).

	Coefficient	*P*-value	95% CI lower	95% CI upper	% Total effect^a^
Practice Visibility—X; Psychological Safety—M; Depersonalization—Y					
Total Effect (X → Y)	−0.15	0.001	−0.23	−0.06	
Mediated Direct Effect (X → Y)	−0.08	>0.05	−0.16	−0.01	
**Indirect Effect (X →M→ Y)**^a^	**−0.07**	**>0.05**	**−0.11**	**−0.04**	**47%** ^b^
X → M (Path a)	0.34	0.001	0.25	0.42	
M → Y (Path b)	−0.21	0.001	−0.30	−0.13	
Independent Practice and Support—X; Psychological Safety—M; Depersonalization—Y					
Total Effect (X → Y)	−0.22	0.001	−0.30	−0.14	
Mediated Direct Effect (X → Y)	−0.15	0.001	−0.24	−0.06	
**Indirect Effect (X →M→ Y)**^a^	**−0.07**	**0.001**	**−0.11**	**−0.04**	**32%** ^b^
X → M (Path a)	0.38	0.001	0.30	0.46	
M → Y (Path b)	−0.18	0.001	−0.27	−0.09	
NP Administration Relations—X; Psychological Safety—M; Depersonalization—Y					
Total Effect (X → Y)	−0.24	0.001	−0.32	−0.16	
Mediated Direct Effect (X → Y)	−0.17	0.001	−0.26	−0.09	
**Indirect Effect (X →M→ Y)**^a^	**−0.07**	**0.001**	**−0.11**	**−0.03**	**29%** ^b^
X → M (Path a)	0.38	0.001	0.29	0.47	
M → Y (Path b)	−0.17	0.001	−0.26	−0.09	
NP Physician Relations—X; Psychological Safety—M; Depersonalization—Y					
Total Effect (X → Y)	−0.17	0.001	−0.26	−0.09	
Mediated Direct Effect (X → Y)	−0.09	0.05	−0.17	0.00	
**Indirect Effect (X →M→ Y)**^a^	**−0.09**	**0.05**	**−0.13**	**−0.04**	**52%** ^a^
X → M (Path a)	0.42	0.001	0.34	0.50	
M → Y (Path b)	−0.20	0.001	−0.29	−0.11	

Abbreviation: NP, nurse practitioner.

All effects adjusted for practice setting, hours worked, and job tenure.

^a^The indirect effect is the key effect of interest. It indicates how much of the total effect of the respective work environment factor on depersonalization is due to the mediating effect of psychological safety.

^b^Proportion of the total effect that is mediated by psychological safety (indirect effect/total effect).

## Discussion

In this study, we describe the work environment, psychological safety, and burnout in a sample of practicing NPs in California (*n* = 621) and investigated the potential mediating effect of psychological safety on the relationship between 4 factors in the work environment and 2 indicators of burnout.

### Factors in the work environment associated with burnout, mediated by psychological safety

To better understand the impact of work environment on burnout, we examined work-related covariates and 4 factors in the work environment and their associations with burnout and psychological safety (Table 2). Job tenure was associated with higher levels of perceived psychological safety and lower burnout. While others have found higher levels of psychological safety within the first year of practice and becoming less over time,^38^ our findings highlight that, for NPs, psychological safety may change over time as they develop practice confidence and social capital. A likely explanation for this is that, with time, NPs develop increased competency and confidence in their practice,^39^ both of which are behaviors associated with speaking up.^40^ All work factors were negatively associated with EE and DE burnout subscale measures and positively associated with psychological safety. Last, our mediation analysis indicated that psychological safety partially mediates the relationship between factors in the work environment and both EE and DE (*P* < .01).

In our study, burnout was negatively associated with PV, or the degree to which NP role clarity and role understanding are perceived within the organization; in contrast, psychological safety was positively associated with PV. Research has shown that role clarity is vital for effective teamwork because it promotes coordination and adaptability needed for successful outcomes.^30^ When role clarity is absent, more cognitive resources are needed to negotiate uncertainty in role function, avoid conflict, and ensure job security, all of which are associated with professional burnout^41^ and turnover in NPs.^39^ Additionally, existing literature indicates that uncertainty and job insecurity are associated with defensive decision-making, defined as decisions made for self-protection rather than decisions that are best for the patient or organization; however, in the presence of psychological safety, defensive decision-making is reduced.^42^ While these studies were not focused on NPs, our findings highlight the importance of psychological safety in enhancing role clarity and practice visibility. Researchers have also found that speaking up, a component of psychological safety, is associated with a more caring work environment^19^ and less burnout.^20^ Our findings and others underscore the need for implementing organizational policies and team practices that promote psychological safety and enhance NP role clarity within the organization.

Independent Practice and Support, a measure to estimate NP perception of practice autonomy and support for patient care management, was negatively associated with burnout. Independent practice autonomy is driven by workplace policies and structural supports that empower NPs and promote control over work tasks and workload.^9^ Researchers have documented that NP autonomy at work is not only associated with resilience^43^ but is also a key element of healthy work environments.^44^ When autonomy is undermined, one's professional role identity is threatened and self-preservation and emotional exhaustion ensue. Similar to other studies of psychological safety,^20^ we found a positive relationship between independent practice autonomy and psychological safety, suggesting that having a voice and autonomy at work may improve work well-being as internal cognitive resources are used for work success rather than self-preservation.^41^

We also assessed work relationships. NP-PR assessed the degree to which the NP feels trusted and valued by physicians and the extent to which physicians seek out NP expertise. We found that higher NP-PR scores were associated with lower burnout scores, suggesting that work well-being is promoted when physician colleagues trust and value NP clinical decision-making. We also found that job tenure and psychological safety were associated with improved NP-PR. Our findings highlight the protective nature of positive working relationships between NPs and physicians and support workplace policies where open communication and speaking up are valued and without fear of retribution.^45^ Hierarchical relations are historically endemic to health care, making it difficult to speak up across professional roles; workplace policies and leader behaviors that promote psychological safety are shown to improve engagement, support learning at work, and improve patient outcomes.^45,46^ Interventions focused on leadership development, communication, and team-building could support NP physician relations, well-being, psychological safety, and patient care outcomes.^16^

Similar to prior studies, participants' perceptions of NP-AR ranked lowest among the 4 work environment subscale factors.^47^ This subscale estimates the degree to which the NP perceives administrative procedures as supportive and that value and respect the NP role. Nurse Practitioner–Administration Relations had the strongest negative association with emotional exhaustion, highlighting the potential negative effects of administrative policies and procedures that undermine NP trust in the organization. Clinician mistrust and lack of confidence in administrators has emerged as a critical issue associated with burnout^1,48^ and highlights the need for systemic reform and realignment toward worker safety and well-being.

### Psychological safety as a partial mediator of burnout

In our study, psychological safety partially mediated the relationship between the 4 work environment factors and EE and DE (*P* < .01). In our analyses, psychological safety emerged as a previously unrecognized mechanism that partly accounts for the impact of a positive work environment on EE and DE.^49^ The inverse relationship between factors in the work environment and burnout scores is partly attributable to psychological safety enabled by a positive work environment. This suggests that the benefits of improving the work environment for NPs may go beyond burnout mitigation and additionally include enhanced psychological safety and its associated benefits. These mediating pathways create opportunities for designing targeted interventions aimed at changes in the work environment to address both burnout and psychological safety simultaneously.

### Implications for practice and policy

Psychological safety is a key metric used to evaluate safety culture.^50^ Patient safety culture has historically been looked at as a measure associated with patient safety and outcomes, yet studies have found associations between safety culture and burnout^1,51,52^ and recent guidance from the World Health Organization applies safety culture to the protection of health workers.^53^ Our findings suggest that policymakers and health systems leaders considering or implementing health system well-being initiatives should consider adopting psychological safety as a metric for assessing the health of the work environment.

A culture of safety is built upon trust and collaboration among clinicians and management^54^ and requires the promotion of psychological safety where NPs can report concerns and errors and ask for help without fear of retaliation. Given the direct, as well indirect, effects of psychological safety on burnout, policies should focus simultaneously or in tandem on structures and practices that promote work environment as well as psychological safety. For instance, polices to enhance NP autonomy could be accompanied by policies that encourage inclusive leadership behaviors that foster psychological safety. Psychological safety is promoted by leader behaviors that foster a culture of inclusion where NPs are valued and involved at all levels of quality improvement.^46,55^ Investing in and developing effective leadership across all health worker groups is needed to promote common language and values alignment toward psychological safety. Such leader skills include confidence in communication and team dynamics,^56^ managing abuse of power and social influence,^57^ and promoting decision-making at the lowest levels of the organization.^49^ Instituting organizational policies that value and promote speaking-up behaviors for all individuals promotes safety culture and reduces the impact of traditional social hierarchies.^57^

### Limitations and implications

This study has several limitations that should be acknowledged. First, the cross-sectional design of this study restricts causal interpretations. Another limitation is the potential for nonresponse bias. The parent study surveyed participants from 9 distinct regions in California; however, there is a risk that the findings may not be fully representative of the entire population. In addition, nonresponse bias could occur if the individuals who did not participate differ significantly from those who did in terms of their experiences of burnout and work environment perceptions. For example, NPs with higher levels of burnout or those working in less-supportive environments may have been less likely to respond, possibly leading to an underestimation of the prevalence of burnout and the challenges within the NP work environment. To mitigate this, we used stratified random sampling and multiple modes of data collection and offered a nominal gift card for participation. We also conducted a comparison between groups who responded to over 70% of survey questions and those who did not and found no statistical difference. Our study was conducted in California, a geographic region where NP practice is restricted, thus potentially limiting the generalizability of findings to other states in the United States where NP practice is not restricted or with other contexts and roles. Our study findings may have been impacted by the COVID-19 pandemic, both due to high burnout and lower response rates to surveys during the pandemic.^58^ Future research should use longitudinal approaches and consider diverse geographical locations where NPs practice to enhance the generalizability of results. Long-term studies could shed light on the evolution of these factors, especially considering changing NP SOP regulations, enabling a deeper understanding and evidence for causal relationships.

## Conclusion

This study highlights the potential role of psychological safety in NP burnout prevention and mitigation. Our findings highlight the interconnected relationships between psychological safety, the NP work environment, and burnout. The findings also underscore the need for multifaceted interventions to address individual well-being, patient and clinician safety, and organizational culture. By fostering psychologically safe environments and tailoring interventions to specific practice settings, health care organizations can contribute to a more resilient, satisfied, and effective workforce that will ultimately improve patient outcomes and the overall quality and safety of health care delivery.

## Supplementary Material

qxae091_Supplementary_Data
