# Supplementary material for: Psychological safety is associated with better work environment and lower levels of clinician burnout
Source: Health Aff Sch. 2024 Jul 17;2(7):qxae091. doi: 10.1093/haschl/qxae091 (PMC11288325; doi:10.1093/haschl/qxae091)
Supplement: qxae091_Supplementary_Data [file qxae091_supplementary_data.zip › Appendix 1 California Survey.docx]

Appendix 1

**Parent Survey and Sample**

The 2022 Survey of California Nurse Practitioners and Nurse Midwives was a 16-page questionnaire including questions about demographics, education and training, practice characteristics, job and career satisfaction, and future practice plans. The statewide survey was conducted by the University of California, San Francisco, with the goal of collecting baseline data before the enactment of legislation that would create a pathway to independent practice in the state.

The survey was fielded from July 2022 and concluded in March of 2023, and at the time of fielding there were 27,649 nurse practitioners (NP) and dually certified nurse midwives (NM) with current California licenses and addresses on record with the California Board of Registered Nursing (BRN). The survey was sent to a stratified random sample of 3,600 NPs and dual licensed NP NMs on record with the BRN. The survey sample was stratified by region to ensure adequate numbers of responses for each region.

The survey was administered online and via a paper survey mailed to participants. The survey was emailed to 492 participants and a paper version was mailed to all those who did not complete the online version as well as the remaining 3,108. Following a modified Dillman approach, emails were followed by two reminder emails and paper surveys were followed by three reminder post-cards. The survey packet (both online and mailed) included information about the study, the survey instrument, a return envelope, and a link to complete the survey online. All participants received a $5 digital gift card, regardless of survey completion.

A total of 993 NPs responded to the parent survey (28% response rate) and of those 46% completed the parent survey online. Each participant was assigned a unique identifier and all identifying information was removed during data cleaning. A total of 96 cases were ineligible due being returned because of incorrect mailing address (n = 95) and a reported death (n = 1). Of the 993 respondents, 757 were currently working in a position requiring NP licensure.
