# Supplementary material for: Psychological safety is associated with better work environment and lower levels of clinician burnout
Source: Health Aff Sch. 2024 Jul 17;2(7):qxae091. doi: 10.1093/haschl/qxae091 (PMC11288325; doi:10.1093/haschl/qxae091)
Supplement: qxae091_Supplementary_Data [file qxae091_supplementary_data.zip › Appendix 2 Study Measures.docx]

Appendix 2

**Study Measures**

**Burnout**

Maslach Burnout Inventory Human Services Survey^1^

The Emotional Exhaustion (9-items) and Depersonalization (5-items) subscales were used for this study and all items were assessed on a 7-point Likert from never (0) to everyday (6). Both scales are scored such that higher scores are indicative of higher levels of each construct and presented using mean scores. Use of the emotional exhaustion raw score (range 0-54) is widely published in the literature and used to define severity of burnout, it is categorized by the publisher as follows: low (0-16), moderate (17-26), and high (27 or over).

Details and sample questions permitted for sharing by the publisher are below.

Subscale 1: Emotional Exhaustion

- I feel emotionally drained from my work

Subscale 2: Depersonalization

- I don’t really care what happens to some clients

Copyright ©1981 Christina Maslach & Susan E. Jackson. All rights reserved in all media. Published by Mind Garden, Inc., www.mindgarden.com

**Psychological Safety**

Psychological Safety Scale^2^

Psychological Safety was assessed using a nine-item single construct scale. Each item was assessed on a 6-point Likert scale from strong disagreement (1) to strong agreement (6), with three of the items being reverse scored. Sample items are listed below with two of the reverse scored items being identified by an (R).

- If you make a mistake on this team, it is often held against you. (R)
- Members of this team are able to bring up problems and tough issues.
- People on this team sometimes reject others for being different. (R)
- It is safe to take a risk on this team.

**Work Environment:**

Nurse Practitioner Primary Care Organizational Climate Questionnaire^3^

The 24-item NP-POCQ scale is a valid and reliable measure of factors in the NP work environment associated with NP practice behaviors and outcomes. The scale has four subscales: professional visibility, independent practice and support, NP-administration relations, and NP-physician relations. For this study, survey items were modified to include NP and NM (nurse midwife), with details and sample questions below. Each item was assessed using a 4-point Likert scale from (1) strong disagreement to (4) strong agreement. Scales were scored such that higher scores indicate more of each construct.

Subscale 1: Professional Visibility (3-items)

- In my organization, the NP/ NM role is well understood
- Administration is well informed of the skills and competencies of NPs/ NMs

Subscale 2: Independent Practice and Support (5-items)

- My organization does not restrict my abilities to practice within my scope of practice
- In my organization, I freely apply all my knowledge and skills to provide patient care
- Physicians and NPs/ NMs have similar support for care management

Subscale 3: NP- Administration Relations (8-items)

- I feel valued by my organization
- Administration treats NPs and physicians equally
- Administration makes efforts to improve working conditions for NPs/ NMs

Subscale 4: NP- Physician Relations (8-items)

- I feel valued by my physician colleagues
- Physicians ask NPs/ NMs for their advice to provide patient care
- Physicians in my practice setting trust my patient care decisions

References

1. Maslach C, Jackson SE. Data from: Maslach Burnout Inventory Human Services Survey 4th Edition. *Mind Garden, Inc*. 1981. *All rights reserved in all media.*

2. Edmondson A. Psychological Safety and Learning Behavior in Work Teams. *Administrative Science Quarterly*. 1999;44(2):350-383. doi:10.2307/2666999

3. Poghosyan L, Ghaffari A, Shaffer J. Nurse practitioner primary care organizational climate questionnaire: Item response theory and differential item functioning. Journal of Clinical Nursing. *Journal of clinical nursing*. 2019;28(15-16):2934-2945. doi:10.1111/jocn.14895
