# Supplementary material for: Psychological safety is associated with better work environment and lower levels of clinician burnout
Source: Health Aff Sch. 2024 Jul 17;2(7):qxae091. doi: 10.1093/haschl/qxae091 (PMC11288325; doi:10.1093/haschl/qxae091)
Supplement: qxae091_Supplementary_Data [file qxae091_supplementary_data.zip › Appendix 3 Correlations.docx]

**Appendix 3.**

**Table 1.** Analysis of study variables by job setting, job tenure, and work hours per week (N=621)

|  | Nurse Practitioner Primary Care Organizational Climate Questionnaire | | | | Psychological Safety Scale | Maslach Burnout Inventory Human Services Survey | | |
| --- | --- | --- | --- | --- | --- | --- | --- | --- |
|  | Practice Visibility | Independent Practice and Support | NP Administrative Relations | NP Physician  Relations |  | Emotional Exhaustion | Depersonalization | |
| Median [IQR]  (min, max)  *[95% CI median]* | 3.0 [2.6, 3.7]  (1.0, 4.0)  [2.9, 3.1] | 3.3 [3.0, 3.9]  (1.0, 4.0)  [3.1, 3.4] | 2.9 [2.3, 3.4]  (1.0, 4.0)  [2.7, 3.0] | 3.2 [2.8, 3.6]  (1.0, 4.0)  [3.1, 3.3] | 4.6 [3.8, 5.2]  (1.0, 6.0)  [4.4, 4.7] | 2.2 [1.1, 3.5]  (0.0, 6.0)  [2.1, 2.3] | 0.6 [0.2, 1.4]  (0.0, 6.0)  [0.6, 0.8] | |
| Setting | Median [IQR] | Median [IQR] | Median  [IQR] | Median [IQR] | Median  [IQR] | Median [IQR] | Median [IQR] | |
| Hospital or Medical Center, n=185 | 3.0 [2.6, 3.4]^b^ | 3.1 [2.8, 3.8]^b^ | 2.8 [2.2, 3.3] | 3.2 [3.0, 3.8] | 4.6 [3.8, 5.2] | 2.0 [1.0, 3.2]^b^ | 0.6 [0.2, 1.2] | |
| Ambulatory Care Setting, n= 301 | 3.0 [3.0, 4.0]^c^ | 3.4 [3.0, 3.9]^c^ | 2.9 [2.5, 3.4] | 3.2 [2.8, 3.6] | 4.6 [3.8, 5.2] | 2.3 [1.3, 3.6]^c^ | 0.6 [0.2, 1.6] | |
| Long-term Care & Home Health, n=26 | 3.0 [2.5, 3.7] | 3.1 [2.6, 3.8] | 2.7 [2.3, 3.1] | 3.0 [2.6, 3.9] | 4.1 [3.5, 4.9] | 2.1 [0.8, 3.6] | 0.6 [0.2, 1.5] | |
| Other, n= 109 | 3.0 [2.6, 3.7] | 3.4 [3.0, 3.9] | 2.8 [2.2, 3.4] | 3.2 [3.0, 3.6] | 4.4 [3.7, 5.1] | 2.2 [1.1, 3.7] | 0.6 [0.2, 1.2] | |
| *p-value* ^a^ | .02 | .01 | .14 | .85 | .39 | .04 | .42 | |
|  | *r (p-value)* | | | | | | |  |
| Job Tenure (yrs) | .08 (.04) | .15 (<.001) | .04 (.34) | .20 (<.001) | .17 (<.001) | -.09 (.03) | -.12 (.004) | |
| Work Week (hrs) | -.08 (.06) | -.08 (.04) | -.02 (.65) | -.06 (.17) | -.08 (.06) | .17 (<.001) | .13 (.002) | |

Abbreviations: IQR, interquartile range; Min, minimum; Max, maximum; CI, confidence interval; yrs, years; hrs, hours.

^a^ANOVA transformed scores

^b & c^ post-hoc tests of difference, Dunnett’s C, p < .001
