# Supplementary material for: Psychological safety is associated with better work environment and lower levels of clinician burnout
Source: Health Aff Sch. 2024 Jul 17;2(7):qxae091. doi: 10.1093/haschl/qxae091 (PMC11288325; doi:10.1093/haschl/qxae091)
Supplement: qxae091_Supplementary_Data [file qxae091_supplementary_data.zip › Appendix 4 Figures.docx]

Appendix 4.

**Figure 1. Mediation Effect of Psychological Safety on the direct effect of Work Environment Factor: NP Physician Relations with Emotional Exhaustion (n= 621)**

Indirect effect: β = -.10*

**% Total Effect = 37%**

NP Physician Relations

MBI: Emotional Exhaustion

Mediated Direct effect: β = -.18*

Total (Unmediated) effect: β = -.27*

Psychological Safety

β = -.23*

β = .42*

*p < .0001

**Figure 2.** **Mediation Effect of Psychological Safety on the direct effects of Work Environment Factor: Independent Practice and Support with Depersonalization (n= 621)**

Independent Practice and Support

MBI: Depersonalization

Psychological Safety

β = -.18*

β = .38*

Indirect effect: β = -.07*

**% Total Effect = 32%**

*p < .001

Mediated Direct effect: β = -.15*

Total (Unmediated) effect: β = -.22*
